# Analysis of the *Listeria monocytogenes* Population Structure among Isolates from 1931 to 2015 in Australia

**DOI:** 10.3389/fmicb.2017.00603

**Published:** 2017-04-06

**Authors:** Amy V. Jennison, Jesse J. Masson, Ning-Xia Fang, Rikki M. Graham, Mark I. Bradbury, Narelle Fegan, Kari S. Gobius, Trudy M. Graham, Christine J. Guglielmino, Janelle L. Brown, Edward M. Fox

**Affiliations:** ^1^Public Health Microbiology, Public and Environmental Health, Queensland Health, Forensic and Scientific Services, BrisbaneQLD, Australia; ^2^Commonwealth Scientific and Industrial Research Organisation – Agriculture and Food, WerribeeVIC, Australia; ^3^Commonwealth Scientific and Industrial Research Organisation – Agriculture and Food, SydneyNSW, Australia

**Keywords:** *Listeria monocytogenes*, serotype, MLST, *inlA*, SNP typing

## Abstract

Listeriosis remains among the most important bacterial illnesses, with a high associated mortality rate. Efforts to control listeriosis require detailed knowledge of the epidemiology of the disease itself, and its etiological bacterium, *Listeria monocytogenes*. In this study we provide an in-depth analysis of the epidemiology of 224 *L. monocytogenes* isolates from Australian clinical and non-clinical sources. Non-human sources included meat, dairy, seafood, fruit, and vegetables, along with animal and environmental isolates. Serotyping, Multi-Locus Sequence Typing, and analysis of *inlA* gene sequence were performed. Serogroups IIA, IIB, and IVB comprised 94% of all isolates, with IVB over-represented among clinical isolates. Serogroup IIA was the most common among dairy and meat isolates. Lineage I isolates were most common among clinical isolates, and 52% of clinical isolates belonged to ST1. Overall 39 STs were identified in this study, with ST1 and ST3 containing the largest numbers of *L. monocytogenes* isolates. These STs comprised 40% of the total isolates (*n* = 90), and both harbored isolates from clinical and non-clinical sources. ST204 was the third most common ST. The high prevalence of this group among *L. monocytogenes* populations has not been reported outside Australia. Twenty-seven percent of the STs in this study contained exclusively clinical isolates. Analysis of the virulence protein InlA among isolates in this study identified a truncated form of the protein among isolates from ST121 and ST325. The ST325 group contained a previously unreported novel mutation leading to production of a 93 amino acid protein. This study provides insights in the population structure of *L. monocytogenes* isolated in Australia, which will contribute to public health knowledge relating to this important human pathogen.

## Introduction

The burden of foodborne disease in Australia has been estimated at an annual cost of AUD$1.2 billion, comprising approximately 5.4 million cases of disease (The OzFoodNet Working Group, 2012). Australia is divided into eight different states or territories, each with its own health department coordinating surveillance for between 10 and 15 foodborne diseases, including listeriosis. As in many other countries, incidence rates of listeriosis are generally far lower than other common foodborne illness, such as campylobacteriosis or salmonellosis, with the rate varying between 0.3 and 0.4 cases per 100,000 since 2008, with most cases/highest rates occurring in age groups over the age of 60 (Rosen, 2002; The OzFoodNet Working Group, 2012). A similar association with high mortality rates is also shared by other countries, with 20–30% of hospitalized cases of invasive listeriosis resulting in fatality (Dalton et al., 2011; Scallan et al., 2011; The OzFoodNet Working Group, 2012). Recent evidence suggests that this burden may be higher, particularly for pregnant women or patients with neurolisteriosis (Charlier et al., 2017).

In addition to the public health burden, the associated costs to the food industry are high – *Listeria monocytogenes* is the main contaminant linked to food recalls in Australia due to microbial contamination, with 45% of these recalls from 2005 through to 2014 due to this organism (based on FSANZ^1^ data); where ready-to-eat (RTE) meat products represented the largest group of recalled products.

In efforts to address these public health and economic costs, countries have employed complex surveillance systems designed to provide knowledge of the epidemiology and population dynamics of *L. monocytogenes*, and to improve detection and response to associated outbreaks of disease (Kirk et al., 2008; Wang et al., 2009; Fox et al., 2012). OzFoodNet was established in Australia in 2000, with government-funded epidemiologists appointed to states and territories to improve surveillance of foodborne disease (Kirk et al., 2008). From 2010 onward, OzFoodNet has enhanced national surveillance of listeriosis, and now collects additional data on invasive *L. monocytogenes* isolates, incorporating molecular sub-typing data to facilitate rapid and precise identification of disease clusters (The OzFoodNet Working Group, 2012). Although recent studies have yielded detailed insights into the population distribution of *L. monocytogenes* globally as well as source associations of important subgroups, such as the over-representation of sequence type (ST) 121 in food sources or of clonal complex (CC) 1 in clinical cases, this data is lacking in the context of Australia (Chenal-Francisque et al., 2011; Maury et al., 2016; Moura et al., 2016).

An important component of understanding the epidemiology of foodborne disease is knowledge of the occurrence and molecular ecology of strains isolated from food products, and indeed the food chain as a whole (Fox et al., 2012). Such information can facilitate insights into the distribution of certain strains within different food chains or environments, and enable relevant associations to be made. Temporal analysis of both clinical and non-clinical surveillance data can allow monitoring of the occurrence of individual strain sub-types or epidemic clones over time, and provides improved understanding of potential risk of disease, and where corrective efforts may be directed. Recent studies have provided insights into global, continental and/or national trends in this area, such as the association of ST121 to food sources, the predominance of CC2 and CC1 globally and association of CC1 with outbreaks of disease, or the dominance of the ST328 subgroup in India (Chenal-Francisque et al., 2011; Haase et al., 2013; Yin et al., 2015; Barbuddhe et al., 2016; Maury et al., 2016). In addition to this, genomic analysis can provide insights into characteristics such as strain virulence. The invasion protein InlA, for example, plays a key role in invasive listeriosis by mediating translocation across the intestinal epithelium (Schäferkordt and Chakraborty, 1997). Mutations in the *inlA* gene have been shown to impact pathogenesis, with premature stop codons (PMSCs) leading to reduced invasion of the infected host (Nightingale et al., 2005; Chen et al., 2011).

Previous studies have reported on the incidence of listeriosis across Australia, and identified risk groups, outbreaks, and risk factors associated with the disease (Dalton et al., 2011; The OzFoodNet Working Group, 2012). In this study, we present molecular typing analyses of *L. monocytogenes* isolated from clinical, environmental, and food sources. Data has been interrogated to identify the prevalent STs among the *L. monocytogenes* population in Australia, and dominant strains have been identified, including their associated food chains and links to clinical illness. This study provides knowledge about *L. monocytogenes* across clinical and non-clinical settings in Australia, and together with previous studies (Dalton et al., 2011; The OzFoodNet Working Group, 2012; Kwong et al., 2016) provides new insights into the epidemiology of listeriosis in Australia and the organism’s associated genetic traits.

## Materials and Methods

### Isolates Included in this Study

This study utilized molecular sub-typing data generated from 224 *L. monocytogenes* isolates, sourced from clinical (*n* = 52), food (*n* = 136), animal/environmental sources (*n* = 33), and source unknown (*n* = 3). Clinical isolates include all notified cases from the State of Queensland from the years 2012 to 2015 (*n* = 39), two additional Queensland isolates (1 each from 2009 to 2010) and additional isolates from New South Wales, South Australia and Victoria (*n* = 5, *n* = 4, and *n* = 1, respectively). Food isolates originated from dairy (*n* = 59), meat (*n* = 51), vegetable (*n* = 4), seafood (*n* = 4), or multiple/unknown matrices (*n* = 18). Food isolates included those collected from the States of Victoria, Queensland, New South Wales, Western Australia, Tasmania, and South Australia (*n* = 67, 34, 16, 13, 4, and 2, respectively). The 33 animal or environmental isolates included 24 animal, 6 unknown food processing environment, and 3 natural environment. Numbers of isolates by year were: 2015, *n* = 16; 2014, *n* = 37; 2013, *n* = 36; 2012, *n* = 20; 2011, *n* = 11; 2010, *n* = 11; 2009, *n* = 13; ≤2008, *n* = 80. Isolates included those from the CSIRO culture collection (*n* = 87), the Queensland Health culture collection (*n* = 79), and isolates from Australia (*n* = 58) detailed in previous studies (Nilsson et al., 2012; Haase et al., 2013). Additional isolate information is contained in **Supplementary Table S1**.

### Serotyping

Molecular determination of serotype was performed using either multiplex PCR (Doumith et al., 2004) or *in silico* analysis of whole genome sequencing data. Isolates were divided into the following groupings: IIA, 1/2a or 3a; IIB, 1/2b or 3b or 7; IIC, 1/2c or 3c; IVA, 4a or 4c; and IVB, 4b or 4d or 4e.

### Multi-Locus Sequence Typing (MLST)

Previously described Multi-Locus Sequence Typing (MLST) data were available for 58 isolates (Nilsson et al., 2012; Haase et al., 2013); the STs of the other 166 isolates were determined using the seven housekeeping gene targets and primers, as previously described (Ragon et al., 2008). *In silico* analysis was performed on genome assemblies generated using the Illumina MiSeq platform (Illumina, San Diego, CA, USA) or the Ion Torrent platform (Life Technologies, USA). Consensus sequences for the housekeeping genes (*abcZ, bglA, cat, dapE, dat, ldh*, and *lhk*) were extracted for each isolate (Ridom SeqSphere+, Ridom GmbH). All phylogenetic analyses were carried out using BioNumerics (v7.5; Applied Maths, Sint-Martens-Latem, Belgium). Minimum spanning trees were generated using minimum spanning tree from categorical data analysis, including partitioning analysis to identify CCs. CCs were assigned to groups with single locus variants.

### *inlA* Sequence and Phylogenetic Analysis

The *inlA* gene sequence was extracted from the *de novo* assemblies of the 166 sequenced isolates, using Geneious (v9.0.5) software (Kearse et al., 2012). These were classified into groups and aligned in Geneious using the translation alignment algorithm (global alignment with Blosum62 cost matrix), with each group having a unique *inlA* sequence (**Supplementary Figure S1**). Phylogenetic analysis (1000 bootstraps) was then performed on the aligned sequences using SplitsTree software (Huson, 1998). Translated protein sequences were derived from the *inlA* gene sequence of each isolate, and examined for PMSCs.

### SNP Typing

The 166 sequenced isolates were variant called by mapping the reads to the appropriate reference strain (IIA, NC_022568.1; IIB, NC_018587.1; IIC, NC_017546; IVB, NC_019556.1) using snippy^2^ with the default settings of minimum 10x coverage and 90% minimum proportion of reads differing to reference. Core single nucleotide polymorphism (SNP) alignments were used for tree generation using the PHYML tree builder (Hasegawa-Kishino-Yano substitution model with 1,000 bootstraps) in the Geneious software package; (Guindon and Gascuel, 2003; Kearse et al., 2012).

### Statistical Analysis

Statistical analysis was performed to investigate the association of ST with clinical, dairy food, or meat food sources, using χ^2^ analysis (where *n* ≥ 10 for a subgroup). The significance of over-representation or under-representation was calculated relative to all isolates or lineage (either I or II), as appropriate.

## Results

### Serotyping Analysis

**FIGURE 1:**
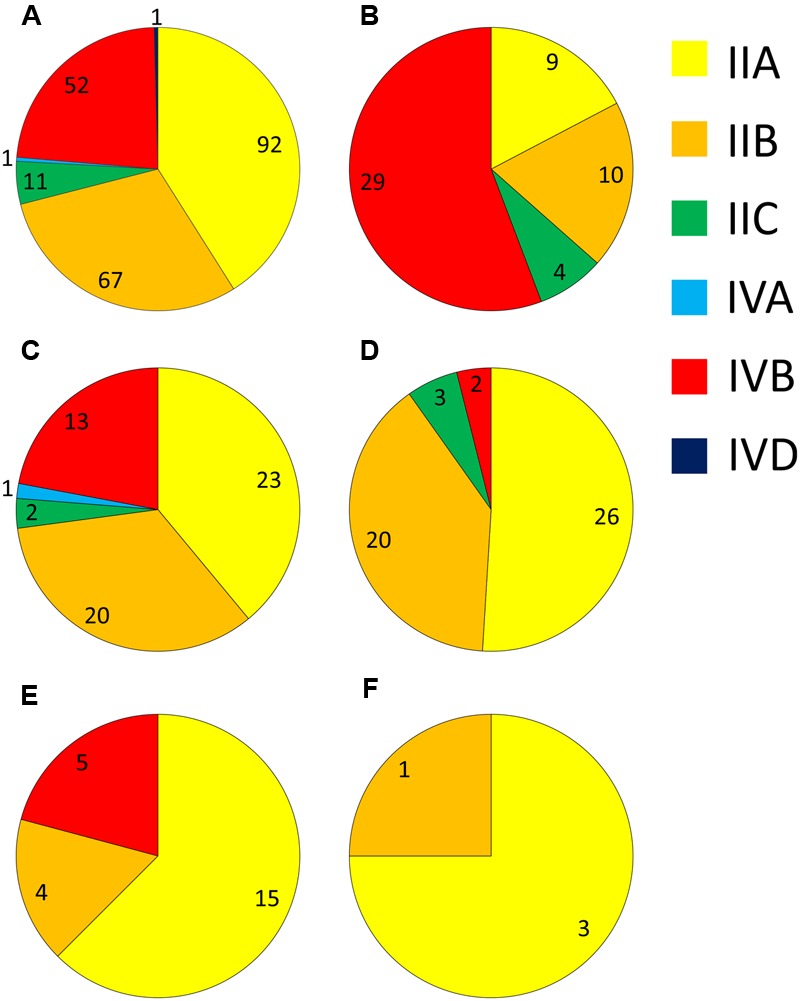
**Distribution of different serogroups among isolates from: (A)** all sources; **(B)** human clinical; **(C)** dairy; **(D)** meat; **(E)** animal; and **(F)** vegetable sources. Numbers of isolates are marked for each serogroup segment.

Analysis of the serotype distribution identified three main serogroups accounting for 94% of isolates – the IIA grouping of 1/2a and 3a (41%), the IIB grouping of 1/2b and 3b serotypes (30%), and the IVB grouping, which included 4b, 4d, and 4e (23%; **Figure 1A**). The IIC grouping of 1/2c and 3c isolates comprised 5%, with IVA and IVD representing the remaining 1%. Serogroup IVB was the predominant group among cases of listeriosis, accounting for 56.6% of clinical isolates, and was significantly over-represented among clinical isolates (*p* < 0.0001; **Figure 1B** and **Supplementary Table S2**). In contrast to this, the occurrence of serogroup IVB isolates was significantly lower among meat category isolates (*p* < 0.001). Serogroup IIA was the largest group among dairy (39%), meat (51%), animal (62.5%) and vegetable (75%) isolates (**Figures 1C–F**, respectively); interestingly among clinical isolates, however, IIA was significantly under-represented (**Supplementary Table S2**). Serogroup IIB was the second most common serogroup identified among isolates from meat, dairy and clinical categories (39.2, 33.9, and 18.9%, respectively), however, there was no significant distribution of this serogroup or serogroup IIC among any of these categories (**Supplementary Table S2**).

### MLST Analysis

**FIGURE 2:**
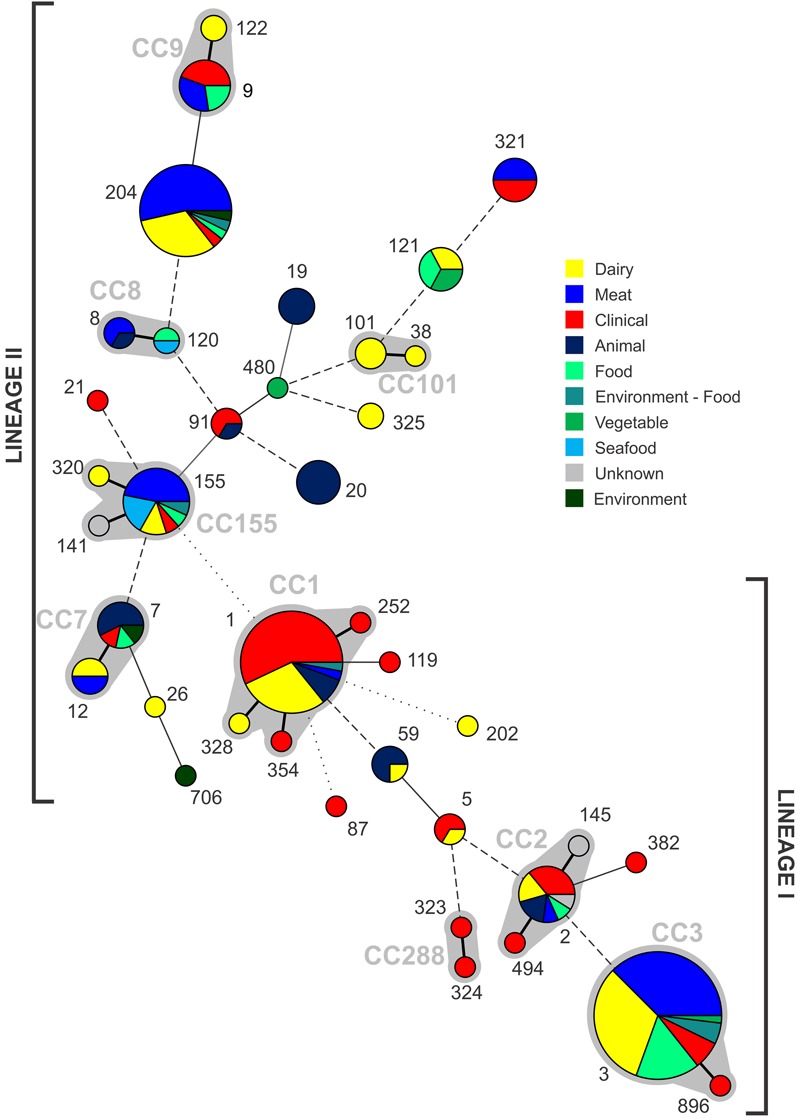
**Distribution of STs identified from each source, with increasing circle size representing a larger number of isolates of that ST.** Clonal complexes are partitioned (gray shading, and indicated by gray text). Connecting lines infer phylogenetic relatedness in terms of number of allelic differences (thick solid, 1; thin solid in partition, 2; thin solid outside partition, 3; broken, 4–6; dotted, no allelic matches). ST202 is lineage III.

**FIGURE 3:**
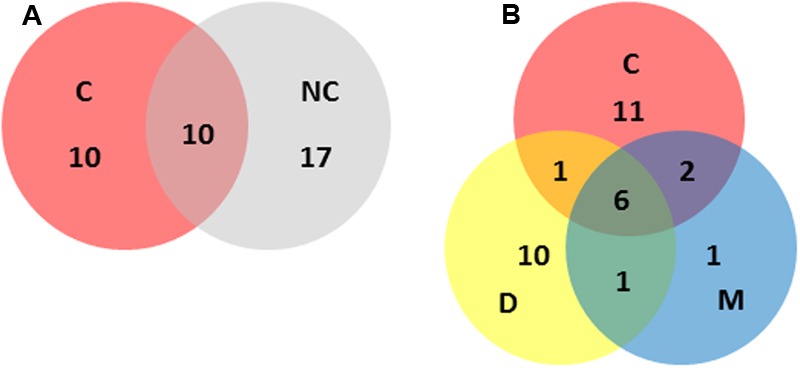
**Distribution of STs across the main categories included in this study (i.e., C, clinical; NC, non-clinical; D, dairy; M, meat).** Numbers represent the number of different STs unique to, or shared by, the relevant categories. **(A)** Clinical versus non-clinical ST distributions. **(B)** Clinical versus dairy versus meat ST distributions.

The 224 isolates differentiated into 39 different STs. The distribution of STs among isolates is presented with respect to their source (**Figure 2**). Seventeen lineage I STs, 22 lineage II STs, and a single lineage III ST (ST202) were identified. Lineage I had the highest proportion of isolates (*n* = 118, 52.7%), with 47.3% (*n* = 106) belonging to lineage II and a single lineage III isolate. ST3 (*n* = 55) and ST1 (*n* = 35) were the largest groupings identified, and comprised 40% of all isolates. Nine CCs were identified, indicated by the gray partitions and labels in **Figure 2**. Lineage II contained the majority of CCs (*n* = 5), with the remaining four CCs comprised of lineage I isolates. A total of 10 STs comprised exclusively clinical isolates: nine were lineage I STs, with a single lineage II ST (**Figure 2**). Of the 39 STs identified, 20 contained clinical isolates (51%, **Figure 3**). In the case of lineage I, 76% of STs contained clinical isolates – this was lower at 32% for lineage II. In contrast to this, of the 17 STs containing only isolates from non-clinical sources, 14 were lineage II, 2 were lineage I, with the remaining being lineage III (the source of the ST141 and ST145 isolates was unknown). The largest single-source categories were ST20 and ST19, comprising 6 and 4 animal isolates, respectively. Three STs only contained dairy isolates: ST101 (*n* = 3), ST122 (*n* = 2), and ST325 (*n* = 2).

With respect to all isolates in this study, χ^2^ analysis (**Supplementary Table S2**) indicated lineage I isolates were over-represented among clinical cases (*p* < 0.001), along with CC1 isolates (*p* < 0.0001). In contrast to this lineage II isolates were under-represented among clinical isolates (*p* < 0.001). While no clear association of dairy isolates with lineage, CC or ST was identified, meat isolates were over-represented among ST204 (*p* < 0.001). In the case of CC3, this subgroup was significantly over-represented among lineage I meat isolates (*p* < 0.001), contrasting to its under-representation among lineage I clinical isolates. There was no significant association of one lineage over another with respect to meat isolates.

### *inlA* Characterization

**FIGURE 4:**
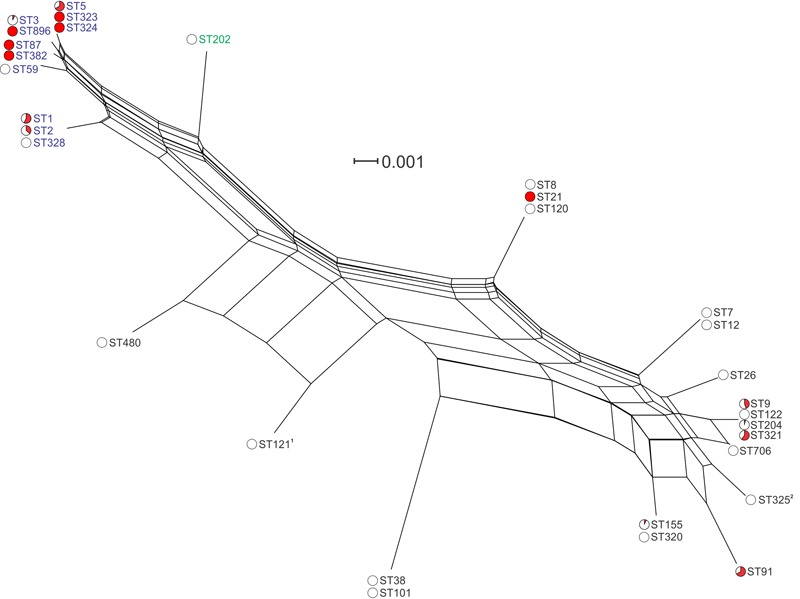
**Phylogenetic analysis of *inlA* gene sequences among isolates in this study.** Lineage I STs are labeled in blue, lineage II STs are labeled in black, and lineage III in green. Red coloring in pie charts indicates the proportion of clinical isolates among all isolates of that ST. ^1^ST121 isolates contained a PMSC at AA^492^; ^2^ST325 isolates contained a PMSC at AA^93^.

Analysis of the *inlA* nucleotide sequence of the 166 sequenced isolates identified 17 variants (InlA groups 1–17, **Figure 4** and **Supplementary Figure S1**). This included 143 variable nucleotide positions, with 40 variable amino acid (AA) residues among the corresponding translated protein sequences. Two of the translated protein sequences, InlA group 4 (shared by all ST3 and ST896 isolates analyzed) and InlA group 15 (shared by all ST5, ST323, and ST324 isolates analyzed), were identical, although differing in three nucleotide positions in the gene sequence. Two of the InlA protein sequences, InlA group 10 (identified in all ST121 isolates analyzed) and InlA group 11 (identified in all ST325 isolates analyzed), contained PMSCs. The PMSC mutation in InlA group 10 isolates results in a protein of 492 AA, whereas the PMSC in InlA group 11 yields a 93 AA InlA protein (the full length InlA is 801 AAs). Clustering analysis of *inlA* gene sequences identified four closely related lineage I InlA groups which includes STs 3, 5, 59, 87, 323, 324, 382, and 896 (**Figure 4**). The *inlA* gene varied at just nine nucleotide positions across these groups, and with the exception of InlA group 14 (ST59) all of these groups contained clinical isolates. Analysis of overall variation by lineage shows greater heterogeneity among lineage II InlA alleles relative to lineage I (115 and 20 variable nucleotide positions among isolates of the same lineage, respectively).

### SNP Subtyping

**FIGURE 5:**
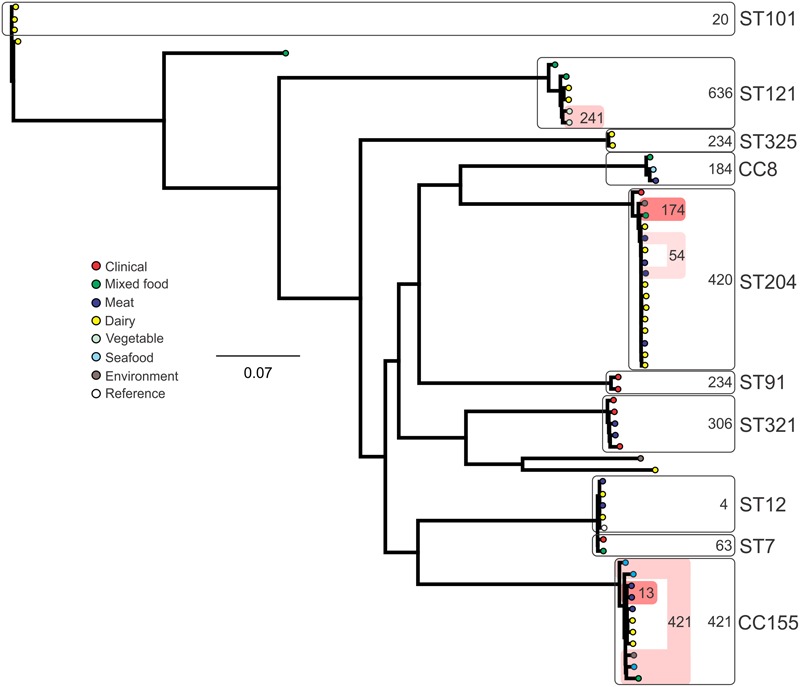
**Phylogenetic analysis of serogroup IIA core genome SNPs.** CCs and STs containing multiple isolates are indicated by labeled rounded rectangles (the CC or ST is labeled on the outside right, and the number of SNPs is indicated by the number on the inside right of the rectangle). Isolates linked by colored shading have a known epidemiological linkage, with the number indicating the highest number of SNPs when comparing isolates in that subset.

**FIGURE 6:**
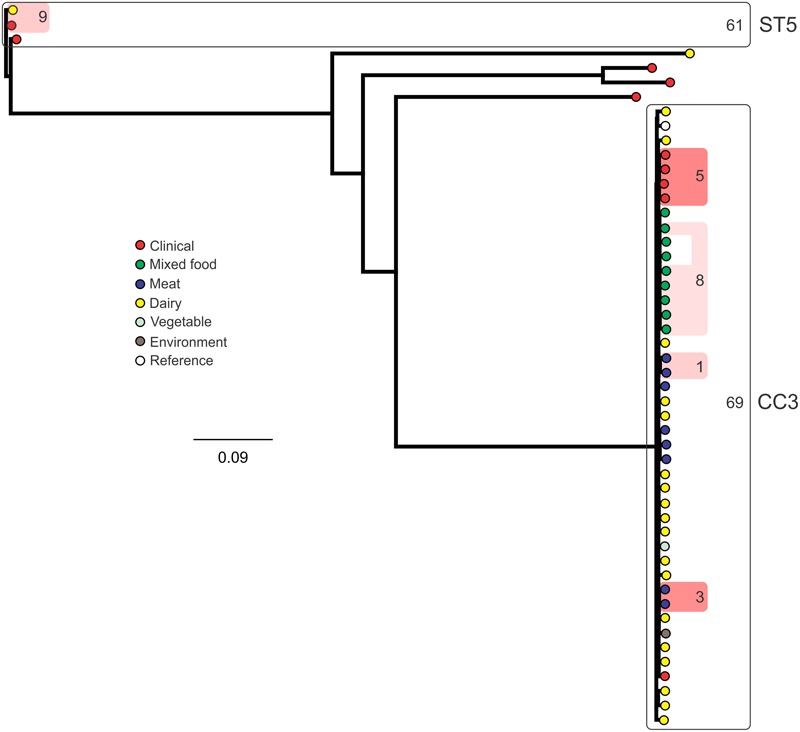
**Phylogenetic analysis of serogroup IIB core genome SNPs.** CCs and STs containing multiple isolates are indicated by labeled rounded rectangles (the CC or ST is labeled on the outside right, and the number of SNPs is indicated by the number on the inside right of the rectangle). Isolates linked by colored shading are epidemiologically linked, with the number indicating the highest number of SNPs when comparing isolates in that subset.

**FIGURE 7:**
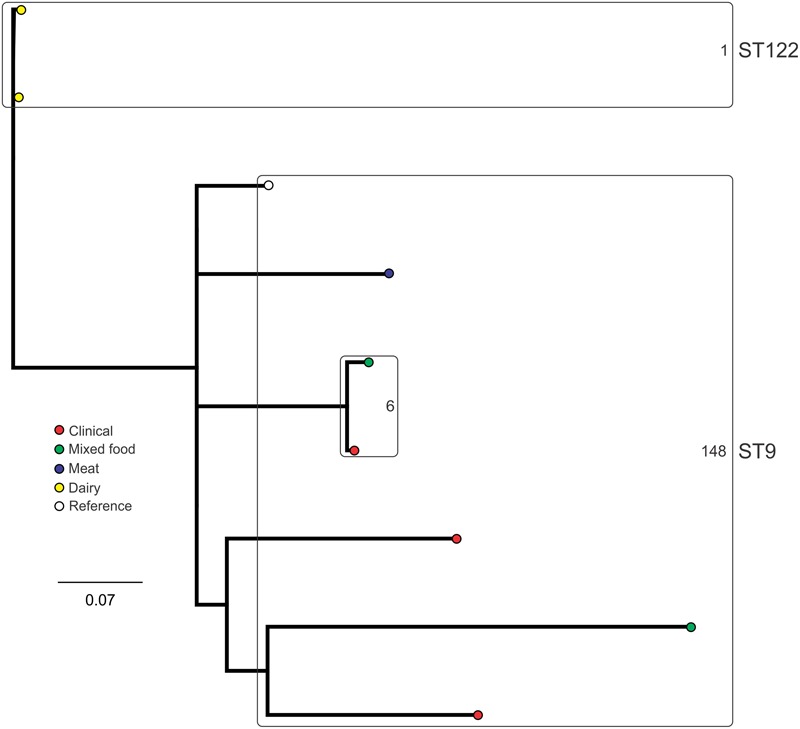
**Phylogenetic analysis of serogroup IIC core genome SNPs.** STs containing multiple isolates are indicated by labeled rounded rectangles (the CC or ST is labeled on the outside right, and the number of SNPs is indicated by the number on the inside right of the rectangle).

**FIGURE 8:**
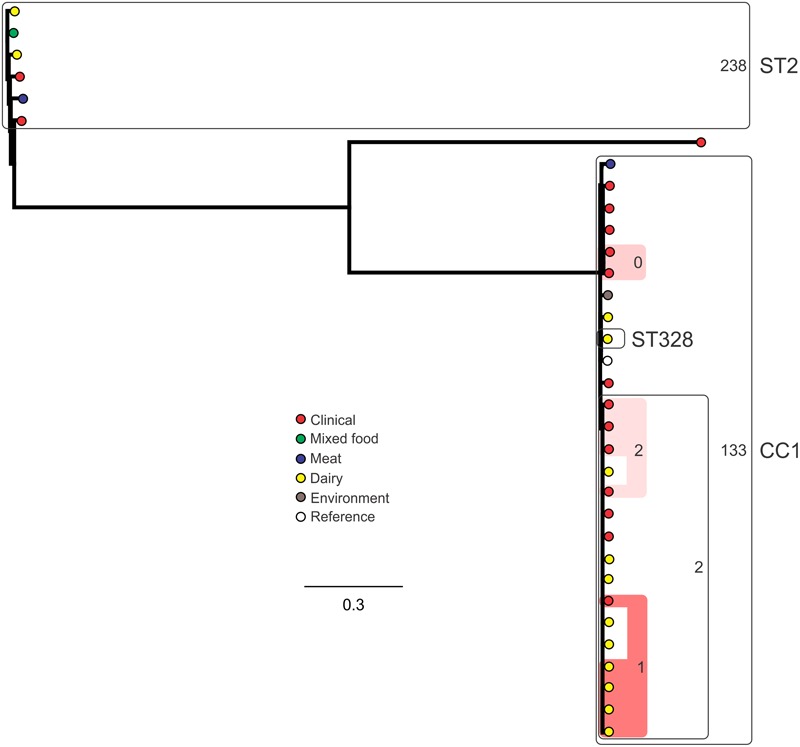
**Phylogenetic analysis of serogroup IVB core genome SNPs.** CCs and STs containing multiple isolates are indicated by labeled rounded rectangles (the CC or ST is labeled on the outside right, and the number of SNPs is indicated by the number on the inside right of the rectangle). Isolates linked by colored shading are epidemiologically linked, with the number indicating the highest number of SNPs when comparing isolates in that subset.

Analysis of genetic diversity among isolates from individual STs showed a high degree of variability (**Figures 5–8**). With the exception of ST12 (four SNPs among the five isolates, which includes the reference strain EGD), serogroup IIA STs were characterized by a high number of SNPs among isolates from individual STs, with ST121, ST155, ST204, and ST321 containing the most SNPs relative to other STs (636, 421, 420, and 306 SNPs, respectively; **Figure 5**). In contrast to this, serogroup IIB STs were highly conserved, as evidenced by only 69 SNPs shared among the 43 CC3 isolates (42 isolates from this study and the reference serotype 1/2b isolate, SLCC2755; **Figure 6**). The small number of serogroup IIC isolates comprised only two STs: ST9 and ST122. Although no epidemiological link was known for the two ST122 isolates, they differed by only a single SNP. Similar to serogroup IIB, the other lineage I serogroup IV was also dominated by a single ST (ST1). Although the overall number of SNPs was higher in CC1 (*n* = 133) relative to CC3 (*n* = 69), groups of highly conserved isolates were noted, including epidemiologically linked and unlinked isolates differing by 0–2 SNPs (**Figure 8**). In contrast to CC1, ST2 isolates were more genetically diverse with 238 SNP loci identified among this group.

## Discussion

Building a detailed understanding of pathogen population demographics, along with clinical and food chain association, is central to building a robust response and control system directed at minimizing the impact of foodborne pathogens to public health. The data presented in this study provides detailed insight into the epidemiology of *L. monocytogenes* in an Australian context by using application of molecular techniques including molecular serotyping, MLST, and analysis of the *inlA* virulence gene marker.

Serotyping was one of the first subtyping methods established to investigate *L. monocytogenes* epidemiology. It has been applied extensively as a rapid means to characterize isolates, with applications in both understanding the relevance of certain serotypes to illness in humans and animals, as well as being employed as a tool to assist in outbreak investigations (Jonquières et al., 1998). Examination of the serotypes of all isolates in this study identified the IIA, IIB, and IVB groupings as the major serotypes (41, 30, and 23%, respectively). This distribution is similar to that observed in other countries, where these groupings, along with the IIC grouping (1/2c and 3c) dominate the *L. monocytogenes* population (Bille and Rocourt, 1996; Wang et al., 2009). Analysis of clinical isolates from Portugal from 1997 to 2004 showed the same top three serogroups, with IVB the most common, followed by IIB and then IIA (72, 18, and 11%, respectively) (Almeida et al., 2010). Of particular note is the absence of serogroup IIC among the Portuguese clinical isolates which comprised 8% of Australian clinical cases in this study. A serogroup hierarchy was noted among clinical isolates in this study with IVB being the most common, followed by IIB then IIA (56, 19, and 17%, respectively). Although lineage I (which includes IVB and IIB serogroups) has previously been identified as over-represented among clinical cases of listeriosis (Sauders et al., 2006), as was seen in this study, it should be noted that lineage II (which includes the IIA and IIC serogroups) has been equally significant in causing infection in some countries (Leong et al., 2015). This demonstrates the importance of lineage II strains in human listeriosis, and although approximately 25% of clinical isolates in this study were lineage II suggesting they remain an important etiological agent of listeriosis in Australia, it is worth noting that serogroup IIA was significantly under-represented among clinical isolates relative to the overall population structure (**Supplementary Table S2**).

The application of MLST to characterize *L. monocytogenes* has yielded many insights into the overall structure of the species population. It has served to establish genetic lineages (I, II, III, and IV), as well as define CCs of highly related *L. monocytogenes* strains, which have been shown to have global distribution (Meinersmann et al., 2004; Ragon et al., 2008). Analysis of the ST distribution among the population (**Figure 2**) identified two main groupings, comprising either lineage I or lineage II isolates. Such definitive separation of lineage I and II has previously been observed using similar analysis of large *L. monocytogenes* ST datasets (Haase et al., 2013). The two largest ST groupings identified, ST3 and ST1 comprising 40% of all isolates (*n* = 90) are both lineage I. These have also been identified globally among the most common STs found in both clinical and non-clinical settings (Ragon et al., 2008; Haase et al., 2013). Clinical isolates were significantly over-represented among the CC1 sub-group, in contrast to an under-representation of CC3 isolates among lineage I clinical isolates (**Supplementary Table S2**). This strong association of CC1 clones with clinical illness has been identified in a recent large study which determined source associations and virulence of *L. monocytogenes* subpopulations (Maury et al., 2016), and it has been hypothesized that this subgroup may have increased virulence relative to others (Henri et al., 2016). That large study also noted ST121 and ST9 as significantly over-represented in food sources, and although all the six ST121 isolates in this study were similarly of food origin, no significant association of CC9 isolates in this study were observed with respect to clinical or food sources (**Supplementary Table S2**).

Many dominant subgroups identified in this study have also been identified as major subgroups in other studies, such as CC1, CC2, CC3, CC9, and CC155 (Chenal-Francisque et al., 2011). In addition to this geographical trends have previously been reported in relation to the *L. monocytogenes* population structure; for example in India a ST328 clone has been identified as widely disseminated among multiple sources including clinical cases (Barbuddhe et al., 2016). Interestingly in this study, ST204 was identified as the third most common ST with isolates from both clinical and a range of non-clinical sources. This is in contrast to other similar studies which have not identified, or recorded at lower abundance, ST204 isolates among *L. monocytogenes* populations from sources outside Australia (Chenal-Francisque et al., 2011; Haase et al., 2013; Unterholzner et al., 2013; Stessl et al., 2014). A notable exception being a recent study on *L. monocytogenes* isolated from various sources in Switzerland reporting ST204 isolates primarily from meat sources (Grohmann et al., 2003). ST204 isolates in this study were identified in a range of sources, including meat, dairy, environmental and clinical samples; in the case of ST204 isolates in this study meat sources were significantly over-represented (**Supplementary Table S2**). Further studies into the genomics of ST204 isolates identified a range of accessory genes involved in different stress response mechanisms among ST204 isolates, often harbored on mobile genetic elements (Fox et al., 2016). A high frequency of plasmid carriage has also been observed (Allnutt et al., 2016). Taken together, this may underlie the capacity for ST204 isolates to colonize a broad range of environmental niches.

Each of the five most common STs (with >10 isolates) identified all contained isolates from a clinical and a variety of non-clinical sources (**Figure 2**). The largest non-clinical ST identified was ST20, which was the 8th largest ST grouping overall, and only contained caprine animal isolates. In addition to ST20, ST19 also comprised isolates exclusively of animal origin, suggesting this may be a niche for these subgroups. Analysis of non-clinical categories identified in the study suggests that there may be a subset of STs linked with dairy sources (ST101, ST122, and ST325), however, a larger dataset is required to further investigate these associations.

Of the STs containing clinical isolates, 50% did not contain isolates from other sources (**Figure 3**). This is consistent with the difficulty in attributing sporadic cases of listeriosis to a food source (Lee et al., 2013). This difficulty is exacerbated by the ubiquitous nature of the organism, and the wide variety of environmental niches it can colonize. A high proportion of STs with raw or RTE meat isolates (80%) also contained isolates from human listeriosis cases. It is noteworthy that meat products comprised the majority of food recalls due to *L. monocytogenes* contamination across Australia during the 2005–2014 period, with Food Standards Australia New Zealand (FSANZ) recall data showing *L. monocytogenes* was responsible for 45% of all food recalls due to microbial contamination in this period^3^. This association is further evidenced in the risk assessment study which suggested processed RTE meats could be responsible for up to 40% of Australian listeriosis cases (Ross et al., 2009). A similar examination of STs with dairy isolates in this study indicated 39% also included clinical isolates, lower than that observed with meat isolates (**Figure 3**). It should be noted, however, that although non-clinical isolates included a diverse representation from multiple States of Australia, the clinical isolates in this study were dominated by those isolates from Queensland. The application of PFGE or whole genome sequencing to epidemiologically linked isolates and analysis with a broader representation of isolates from other Australian States may provide greater insight into strains shared by food and clinical sources. Continued surveillance should include quantitative microbial risk assessment to identify the contribution of different foods to the burden of human infection (Ross et al., 2009). In addition the application of source attribution modeling should be applied. This approach utilizes modeling, such as the Hald Bayesian risk attribution model, to combine highly discriminatory subtyping analysis (e.g., SNP analysis or Pulsed-Field Gel Electrophoresis) with data relating to human sporadic infection and the prevalence of those subtypes in different foods, to identify likely sources (Hald et al., 2004; Little et al., 2010). Such approaches can help identify key points to direct food safety strategies and public health interventions.

Internalin A (InlA) is a virulence protein associated with the invasive form of listeriosis (Schäferkordt and Chakraborty, 1997). This membrane-associated protein binds E-cadherin and facilitates the organism’s entry into intestinal epithelial cells, a key step in the invasive process. Analysis of the *inlA* sequence from isolates in this study identified a heterogeneous nucleotide sequence among isolates, with 17 *inlA* sequence variants showing higher conservation among lineage I isolates (**Figure 4**). This cluster of closely related lineage I isolates was noted with a lower sequence variation relative to other InlA groups (InlA groups 4, 14, 15, and 16; **Supplementary Figure S1**). Seven of the eight STs in this closely related group included clinical isolates. Along with the lineage I InlA group 3 (ST1, ST2, and ST328) these lineage I isolates also clustered separately from other lineages (**Figure 4**). In contrast to lineage I isolates, lineage II isolates showed higher heterogeneity in their InlA sequences with a higher number of alleles showing greater genetic variation. In addition to this only 36% of these alleles (4/11) contained clinical isolates, suggesting this higher divergence in InlA tends to be associated with isolates from non-clinical sources. Previous studies have identified PMSCs in *inlA*, leading to truncated or secreted forms of the protein (Autret et al., 2001; Nightingale et al., 2005). These mutations have been associated with reduced invasiveness of strains harboring them. Typically the same mutations are shared by strains among an individual ST. Of the STs identified in this study, two had PMSCs: all ST121 isolates had a mutation AA^492^ and all ST325 isolates had a mutation at AA^93^ (both of which are lineage II STs). Although the ST121 PMSC mutation has been previously reported, to the authors’ knowledge this is the first report of the novel AA^93^ mutation. No ST121 or ST325 isolates in this study were from clinical sources. Although taken together this data suggests these STs may be less invasive in human patients, further study is required to confirm this.

Single nucleotide polymorphism typing analysis showed a genetically diverse serogroup IIA, with individual STs or CCs often sharing a large number of SNPs (up to 636 in the case of the most diverse ST, ST121). In addition, epidemiologically linked isolates often included a high number of SNP differences (ranging from 13 to 241). In contrast, a high degree of genetic conservation was observed among serogroup IIB isolates, particularly CC3 isolates (**Figure 6**). Serogroup IVB was also dominated by a single CC (CC1), which also included a clinical isolate linked to contaminated dairy products (differing by only a single SNP). The only other clinical case which was associated with a closely related food (also a dairy food) isolate was noted among ST5, although there were nine SNPs in this instance. This number is in agreement with observations in similar studies where epidemiologically linked isolates differed by less than 10 SNPs (Kwong et al., 2016). Among serogroup IIC, a clinical ST9 isolate shared only six SNPs with a food isolates, although no epidemiological link was known in this case to suggest an association. All other epidemiologically linked isolates were either exclusively from foods, or only isolated from clinical cases with no known associated food source. Although this study used SNP typing analysis to examine population structure, further investigations in our laboratory are ongoing into the loci of SNPs in relation to their impact on genetic functionality and associated phenotypes such as virulence and stress resistance.

Surveillance of foodborne disease in Australia has continued to evolve to maintain Australia’s high food safety standards and well-coordinated public health program, notably through the establishment of OzFoodNet in 2000, and the enhanced listeriosis surveillance program implemented in 2010 (The OzFoodNet Working Group, 2012). Integrating detailed analysis of epidemiological data to national, and international, surveillance programs can serve to improve the efficacy and response to threats to human health posed by foodborne pathogens such as *L. monocytogenes*. This study highlights the association of subpopulations of *L. monocytogenes* to different sources in Australia, including those associated with clinical illness, and identifies genetic mutations suggesting attenuated virulence of certain subgroups in humans. The ubiquitous ecology of *L. monocytogenes* presents a significant challenge for source tracking, which is further exacerbated by the long incubation time of disease in patients following consumption of contaminated foods. Surveillance data where epidemiological linkages are known will help further understanding of key transmission routes and high risk foods. Additional analysis also including isolates from samples not highly represented in this study (e.g., fruit, vegetables, and seafood) will increase understanding of the distribution of *L. monocytogenes* subtypes through these different sources. These insights can be utilized to help maintain Australian public health and food safety.

## Author Contributions

AJ, NF, KG, JB, and EF conceived and designed the experiments. N-XF, RG, MB, TG, and CG performed wet laboratory experiments. AJ, JM, JB, and EF conducted bioinformatics analyses. AJ and EF drafted manuscript. All authors read and approved final manuscript and agree to be accountable for all aspects of the work in ensuring that questions related to the accuracy or integrity of any part of the work are appropriately investigated and resolved.

## Conflict of Interest Statement

The authors declare that the research was conducted in the absence of any commercial or financial relationships that could be construed as a potential conflict of interest.

## Footnotes

1 http://www.foodstandards.gov.au/industry/foodrecalls/recallstats/Pages/default.aspx#

2 https://github.com/tseemann/snippy

3 http://www.foodstandards.gov.au/industry/foodrecalls/recallstats/pages/default.aspx

## Supplementary Material

FIGURE S1 **Alignment of each *inlA* gene allele identified among isolates in this study**.

TABLE S1 **Isolates included in this study**.

TABLE S2 **Distribution of major CCs, STs, or serogroups identified in this study from clinical, dairy or meat sources.** The significance of this distribution to each source relative to either the overall population or to the associated lineage is shown, as determined by χ^2^ analysis. Significance thresholds used are: ^∗∗^*p* < 0.00001; ^∗^*p* < 0.001.

## References

[B1] Allnutt T. R. Bradbury M. I. Fanning S. Chandry P. S. Fox E. M. (2016). Draft genome sequences of 15 isolates of *Listeria monocytogenes* Serotype 1/2a, Subgroup ST204. *Genome Announc.* 4 e00935–16. 10.1128/genomeA.00935-16 27609916 PMC5017221

[B2] Almeida G. Morvan A. Magalhaes R. Santos I. Hogg T. Leclercq A. (2010). Distribution and characterization of *Listeria monocytogenes* clinical isolates in Portugal, 1994-2007. *Eur. J. Clin. Microbiol. Infect. Dis.* 29 1219–1227. 10.1007/s10096-010-0988-x 20563829

[B3] Autret N. Dubail I. Trieu-Cuot P. Berche P. Charbit A. (2001). Identification of new genes involved in the virulence of *Listeria monocytogenes* by signature-tagged transposon mutagenesis. *Infect. Immun.* 69 2054–2065. 10.1128/IAI.69.4.2054-2065.2001 11254558 PMC98130

[B4] Barbuddhe S. B. Doijad S. P. Goesmann A. Hilker R. Poharkar K. V. Rawool D. B. (2016). Presence of a widely disseminated *Listeria monocytogenes* serotype 4b clone in India. *Emerg. Microbes Infect.* 5:e55 10.1038/emi.2016.55 PMC4932648 27273224

[B5] Bille J. Rocourt J. (1996). WHO international multicenter *Listeria monocytogenes* subtyping study - rationale and set-up of the study. *Int. J. Food Microbiol.* 32 12. 10.1016/s0168-1605(96)01140-3 8913798

[B6] Charlier C. Perrodeau É Leclercq A. Cazenave B. Pilmis B. Henry B. (2017). Clinical features and prognostic factors of listeriosis: the MONALISA national prospective cohort study. *Lancet Infect. Dis.* 10.1016/S1473-3099(16)30521-7 [Epub ahead of print]. 28139432

[B7] Chen Y. Ross W. H. Whiting R. C. Van Stelten A. Nightingale K. K. Wiedmann M. (2011). Variation in *Listeria monocytogenes* dose responses in relation to subtypes encoding a full-length or truncated internalin A. *Appl. Environ. Microbiol.* 77 1171–1180. 10.1128/AEM.01564-10 21169442 PMC3067222

[B8] Chenal-Francisque V. Lopez J. Cantinelli T. Caro V. Tran C. Leclercq A. (2011). Worldwide distribution of major clones of *Listeria monocytogenes*. *Emerg. Infect. Dis.* 17 1110–1112. 10.3201/eid1706.101778 21749783 PMC3358213

[B9] Dalton C. B. Merritt T. D. Unicomb L. E. Kirk M. D. Stafford R. J. Lalor K. (2011). A national case-control study of risk factors for listeriosis in Australia. *Epidemiol. Infect.* 139 437–445. 10.1017/S0950268810000944 20429970

[B10] Doumith M. Buchrieser C. Glaser P. Jacquet C. Martin P. (2004). Differentiation of the major *Listeria monocytogenes* serovars by multiplex PCR. *J. Clin. Microbiol.* 42 3819–3822. 10.1128/JCM.42.8.3819-3822.2004 15297538 PMC497638

[B11] Fox E. M. Allnutt T. Bradbury M. I. Fanning S. Chandry P. S. (2016). Comparative genomics of the *Listeria monocytogenes* ST204 subgroup. *Front. Microbiol.* 7:2057 10.3389/fmicb.2016.02057 PMC5177744 28066377

[B12] Fox E. M. deLappe N. Garvey P. McKeown P. Cormican M. Leonard N. (2012). PFGE analysis of *Listeria monocytogenes* isolates of clinical, animal, food and environmental origin from Ireland. *J. Med. Microbiol.* 61(Pt 4) 540–547. 10.1099/jmm.0.036764-0 22116984

[B13] Grohmann E. Muth G. Espinosa M. (2003). Conjugative plasmid transfer in Gram-positive bacteria. *Microbiol. Mol. Biol. Rev.* 67 277–301. 10.1128/mmbr.67.2.277-301.2003 12794193 PMC156469

[B14] Guindon S. Gascuel O. (2003). A simple, fast, and accurate algorithm to estimate large phylogenies by maximum likelihood. *Syst. Biol.* 52 696–704. 10.1080/10635150390235520 14530136

[B15] Haase J. K. Didelot X. Lecuit M. Korkeala H. Group L. M. M. S. Achtman M. (2013). The ubiquitous nature of *Listeria monocytogenes* clones: a large-scale multilocus sequence typing study. *Environ. Microbiol.* 16 405–416. 10.1111/1462-2920.12342 24274459

[B16] Hald T. Vose D. Wegener H. C. Koupeev T. (2004). A bayesian approach to quantify the contribution of animal-food sources to human salmonellosis. *Risk Anal.* 24 255–269. 10.1111/j.0272-4332.2004.00427.x 15028016

[B17] Henri C. Félix B. Guillier L. Leekitcharoenphon P. Michelon D. Mariet J.-F. (2016). Population genetic structure of *Listeria monocytogenes* strains as determined by pulsed-field gel electrophoresis and multilocus sequence typing. *Appl. Environ. Microbiol.* 82 5720–5728. 10.1128/aem.00583-16 27235443 PMC5007763

[B18] Huson D. H. (1998). SplitsTree: analyzing and visualizing evolutionary data. *Bioinformatics* 14 68–73. 10.1093/bioinformatics/14.1.68 9520503

[B19] Jonquières R. Bierne H. Mengaud J. Cossart P. (1998). The *inlA* gene of *Listeria monocytogenes* LO28 harbors a nonsense mutation resulting in release of internalin. *Infect. Immun.* 66 3420–3422. 9632615 10.1128/iai.66.7.3420-3422.1998 PMC108362

[B20] Kearse M. Moir R. Wilson A. Stones-Havas S. Cheung M. Sturrock S. (2012). Geneious basic: an integrated and extendable desktop software platform for the organization and analysis of sequence data. *Bioinformatics* 28 13 10.1093/bioinformatics/bts199 PMC3371832 22543367

[B21] Kirk M. D. McKay I. Hall G. V. Dalton C. B. Stafford R. Unicomb L. (2008). Food safety: foodborne disease in Australia: the OzFoodNet experience. *Clin. Infect. Dis.* 47 392–400. 10.1086/589861 18558879

[B22] Kwong J. C. Mercoulia K. Tomita T. Easton M. Li H. Y. Bulach D. M. (2016). Prospective whole-genome sequencing enhances national surveillance of *Listeria monocytogenes*. *J. Clin. Microbiol.* 54 333–342. 10.1128/jcm.02344-15 26607978 PMC4733179

[B23] Lee S. Rakic-Martinez M. Graves L. M. Ward T. J. Siletzky R. M. Kathariou S. (2013). Genetic determinants for cadmium and arsenic resistance among *Listeria monocytogenes* serotype 4b isolates from sporadic human listeriosis patients. *Appl. Environ. Microbiol.* 79 2471–2476. 10.1128/AEM.03551-12 23377929 PMC3623226

[B24] Leong D. Alvarez-Ordóñez A. Zaouali S. Jordan K. (2015). Examination of *Listeria monocytogenes* in seafood processing facilities and smoked salmon in the republic of Ireland. *J. Food Prot.* 78 2184–2190. 10.4315/0362-028X.JFP-15-233 26613913

[B25] Little C. L. Pires S. M. Gillespie I. A. Grant K. Nichols G. L. (2010). Attribution of human *Listeria monocytogenes* infections in england and wales to ready-to-eat food sources placed on the market: adaptation of the hald *Salmonella* source attribution model. *Foodborne Pathog. Dis.* 7 749–756. 10.1089/fpd.2009.0439 20156087

[B26] Maury M. M. Tsai Y.-H. Charlier C. Touchon M. Chenal-Francisque V. Leclercq A. (2016). Uncovering *Listeria monocytogenes* hypervirulence by harnessing its biodiversity. *Nat. Genet.* 48 308–313. 10.1038/ng.3501 26829754 PMC4768348

[B27] Meinersmann R. J. Phillips R. W. Wiedmann M. Berrang M. E. (2004). Multilocus sequence typing of *Listeria monocytogenes* by use of hypervariable genes reveals clonal and recombination histories of three lineages. *Appl. Environ. Microbiol.* 70 2193–2203. 10.1128/aem.70.4.2193-2203.2004 15066813 PMC383165

[B28] Moura A. Criscuolo A. Pouseele H. Maury M. M. Leclercq A. Tarr C. (2016). Whole genome-based population biology and epidemiological surveillance of *Listeria monocytogenes*. *Nature Microbiology* 2 16185 10.1038/nmicrobiol.2016.185 PMC8903085 27723724

[B29] Nightingale K. K. Windham K. Martin K. E. Yeung M. Wiedmann M. (2005). Select *Listeria monocytogenes* subtypes commonly found in foods carry distinct nonsense mutations in *inlA*, leading to expression of truncated and secreted internalin A, and are associated with a reduced invasion phenotype for human intestinal epithelial cells. *Appl. Environ. Microbiol.* 71 8764–8772. 10.1128/AEM.71.12.8764-8772.2005 16332872 PMC1317312

[B30] Nilsson R. E. Latham R. Mellefont L. Ross T. Bowman J. P. (2012). MudPIT analysis of alkaline tolerance by *Listeria monocytogenes* strains recovered as persistent food factory contaminants. *Food Microbiol.* 30 187–196. 10.1016/j.fm.2011.10.004 22265300

[B31] Ragon M. Wirth T. Hollandt F. Lavenir R. Lecuit M. Le Monnier A. (2008). A new perspective on *Listeria monocytogenes* evolution. *PLoS Pathog.* 4:e1000146 10.1371/journal.ppat.1000146 PMC2518857 18773117

[B32] Rosen B. P. (2002). Biochemistry of arsenic detoxification. *FEBS Lett.* 529 86–92. 10.1016/S0014-5793(02)03186-1 12354618

[B33] Ross T. Rasmussen S. Fazil A. Paoli G. Sumner J. (2009). Quantitative risk assessment of *Listeria monocytogenes* in ready-to-eat meats in Australia. *Int. J. Food Microbiol.* 131 128–137. 10.1016/j.ijfoodmicro.2009.02.007 19327859

[B34] Sauders B. D. Durak M. Z. Fortes E. Windham K. Schukken Y. Lembo A. J. (2006). Molecular characterization of *Listeria monocytogenes* from natural and urban environments. *J. Food Prot.* 69 93–105. 16416906 10.4315/0362-028x-69.1.93

[B35] Scallan E. Hoekstra R. M. Angulo F. J. Tauxe R. V. Widdowson M.-A. Roy S. L. (2011). Foodborne illness acquired in the united states–major pathogens. *Emerg. Infect. Dis.* 17 7–15. 10.3201/eid1701.P11101 21192848 PMC3375761

[B36] Schäferkordt S. Chakraborty T. (1997). Identification, cloning, and characterization of the *lma* operon, whose gene products are unique to *Listeria monocytogenes*. *J. Bacteriol.* 179 2707–2716. 9098070 10.1128/jb.179.8.2707-2716.1997 PMC179021

[B37] Stessl B. Fricker M. Fox E. Karpiskova R. Demnerova K. Jordan K. (2014). Collaborative survey on the colonization of different types of cheese-processing facilities with *Listeria monocytogenes*. *Foodborne Pathog. Dis.* 11 8–14. 10.1089/fpd.2013.1578 24138033

[B38] The OzFoodNet Working Group (2012). Monitoring the incidence and causes of diseases potentially transmitted by food in Australia: annual report of the OzFoodNet Network, 2010. *Commun. Dis. Intell. Q. Rep.* 36 E213–41. 23186234 10.33321/cdi.2012.36.17

[B39] Unterholzner S. J. Poppenberger B. Rozhon W. (2013). Toxin–antitoxin systems: biology, identification, and application. *Mob. Genet. Elements* 3:e26219 10.4161/mge.26219 PMC3827094 24251069

[B40] Wang L. Jeon B. Sahin O. Zhang Q. (2009). Identification of an arsenic resistance and arsenic-sensing system in *Campylobacter jejuni*. *Appl. Environ. Microbiol.* 75 5064–5073. 10.1128/aem.00149-09 19502436 PMC2725487

[B41] Yin Y. Tan W. Wang G. Kong S. Zhou X. Zhao D. (2015). Geographical and longitudinal analysis of *Listeria monocytogenes* genetic diversity reveals its correlation with virulence and unique evolution. *Microbiol. Res.* 175 84–92. 10.1016/j.micres.2015.04.002 25912377

