# Supplementary figures and images for: Analysis of the Listeria monocytogenes Population Structure among Isolates from 1931 to 2015 in Australia
Source: Front Microbiol. 2017 Apr 6;8:603. doi: 10.3389/fmicb.2017.00603 (PMC5382192; doi:10.3389/fmicb.2017.00603)

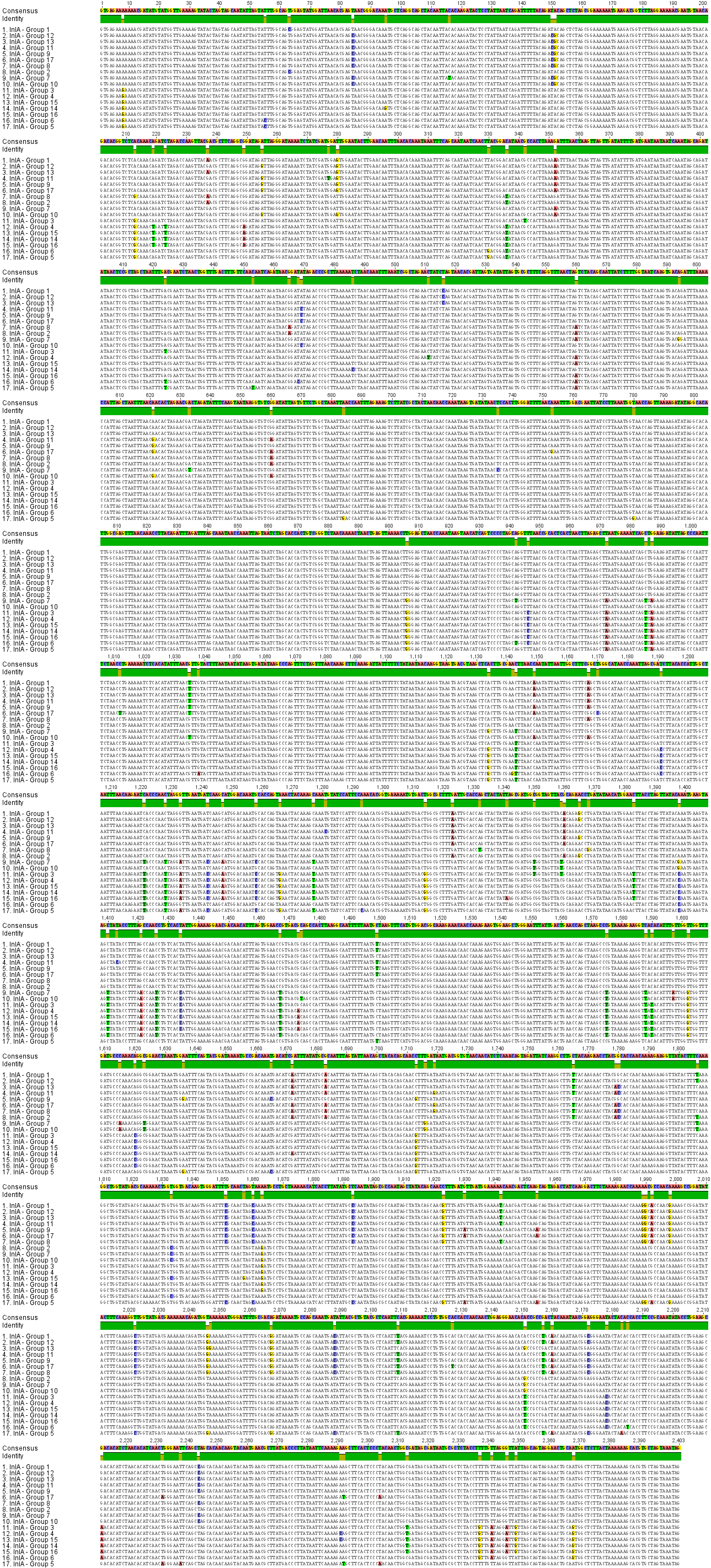

Supplement: FIGURE S1 — Alignment of each inlA gene allele identified among isolates in this study. [file Image_1.TIF]
