# Supplementary material for: Analysis of the Listeria monocytogenes Population Structure among Isolates from 1931 to 2015 in Australia
Source: Front Microbiol. 2017 Apr 6;8:603. doi: 10.3389/fmicb.2017.00603 (PMC5382192; doi:10.3389/fmicb.2017.00603)
Supplement: TABLE S1 — Isolates included in this study. [file Table_1.DOCX]

| **Key** | **Serotype** | **MLST ST** | **Lineage** | **Category** | **Date** | **Isolation context** | **NCBI Accession** |
| --- | --- | --- | --- | --- | --- | --- | --- |
| 2544 | 4b, 4d, 4e | 1 | I | Clinical | 1994 | Clinical case |  |
| 2727 | 4b, 4d, 4e | 1 | I | Meat | 1988 | Product testing | NAVA00000000 |
| 2878 | 4b, 4d, 4e | 1 | I | Dairy | 2013 | Product testing |  |
| 2879 | 4b, 4d, 4e | 1 | I | Dairy | 2013 | Product testing |  |
| 2880 | 4b, 4d, 4e | 1 | I | Dairy | 2013 | Product testing |  |
| 2883 | 4b, 4d, 4e | 1 | I | Dairy | 2013 | Product testing |  |
| 2913 | 4b, 4d, 4e | 1 | I | Dairy | 2013 | Routine surveillance |  |
| 2969 | 4b, 4d, 4e | 1 | I | Dairy | 2012 | Product testing |  |
| 2983 | 4b, 4d, 4e | 1 | I | Clinical | 2012 | Clinical case |  |
| 2986 | 4b, 4d, 4e | 1 | I | Dairy | 2013 | Product testing |  |
| 2991 | 4b, 4d, 4e | 1 | I | Dairy | 2013 | Routine surveillance |  |
| 2993 | 4b, 4d, 4e | 1 | I | Dairy | 2009 | Routine surveillance | LJPF01000000 |
| L4937a | 4b | 1 | I | Animal | 1991 | Animal isolate |  |
| L4937b | 4b | 1 | I | Animal | 1991 | Animal isolate |  |
| L4948a | 4b | 1 | I | Animal | 1991 | Caprine isolate |  |
| QH19 | 4b, 4d, 4e | 1 | I | Dairy | 2013 | Product testing |  |
| QH32 | 4b, 4d, 4e | 1 | I | Environment - Food | 2015 | Routine surveillance |  |
| QH44 | 4b, 4d, 4e | 1 | I | Clinical | 2012 | Clinical case |  |
| QH45 | 4b, 4d, 4e | 1 | I | Clinical | 2013 | Clinical case |  |
| QH48 | 4b, 4d, 4e | 1 | I | Clinical | 2013 | Clinical case |  |
| QH49 | 4b, 4d, 4e | 1 | I | Clinical | 2013 | Clinical case |  |
| QH50 | 4b, 4d, 4e | 1 | I | Clinical | 2013 | Clinical case |  |
| QH52 | 4b, 4d, 4e | 1 | I | Clinical | 2013 | Clinical case |  |
| QH53 | 4b, 4d, 4e | 1 | I | Clinical | 2013 | Clinical case |  |
| QH56 | 4b, 4d, 4e | 1 | I | Clinical | 2014 | Clinical case |  |
| QH57 | 4b, 4d, 4e | 1 | I | Clinical | 2014 | Clinical case |  |
| QH61 | 4b, 4d, 4e | 1 | I | Clinical | 2014 | Clinical case |  |
| QH63 | 4b, 4d, 4e | 1 | I | Clinical | 2014 | Clinical case |  |
| QH68 | 4b, 4d, 4e | 1 | I | Clinical | 2014 | Clinical case |  |
| QH77 | 4b, 4d, 4e | 1 | I | Clinical | 2015 | Clinical case |  |
| QH78 | 4b, 4d, 4e | 1 | I | Clinical | 2015 | Clinical case |  |
| SLCC2534 | 4b | 1 | I | Clinical | 1966 | Clinical case |  |
| SLCC2535 | 4b | 1 | I | Clinical | 1966 | Clinical case |  |
| SLCC4316 | 4b | 1 | I | Clinical | 1975 | Clinical case |  |
| SLCC4317 | 4b | 1 | I | Clinical | 1975 | Clinical case |  |
| 2473 | 4b, 4d, 4e | 2 | I | Dairy | 1998 | Product testing |  |
| 2995 | 4b, 4d, 4e | 2 | I | Dairy | 2009 | Routine surveillance |  |
| 3003 | 4b, 4d, 4e | 2 | I | Food | 2008 | Product testing |  |
| L4939a | 4b | 2 | I | Animal | 1991 | Animal isolate |  |
| L4939b | 4b | 2 | I | Animal | 1991 | Animal isolate |  |
| LM13007 | 4b | 2 | I | Unknown | 1989 | Unknown |  |
| QH58 | 4b, 4d, 4e | 2 | I | Clinical | 2014 | Clinical case |  |
| QH59 | 4b, 4d, 4e | 2 | I | Clinical | 2014 | Clinical case |  |
| QH73 | 4b, 4d, 4e | 2 | I | Clinical | 2015 | Clinical case |  |
| QH9 | 4b, 4d, 4e | 2 | I | Meat | 2013 | Product testing |  |
| SLCC2654 | 4b | 2 | I | Clinical | 1967 | Clinical case |  |
| 2538 | 1/2b, 3b | 3 | I | Dairy | 1994 | Routine surveillance |  |
| 2619 | 1/2b, 3b | 3 | I | Vegetable | 1998 | Product testing |  |
| 2887 | 1/2b, 3b | 3 | I | Dairy | 2007 | Routine surveillance | NAUZ00000000 |
| 2920 | 1/2b | 3 | I | Meat | 2007 | Routine surveillance |  |
| 2926 | 1/2b | 3 | I | Meat | 2007 | Routine surveillance |  |
| 2927 | 1/2b | 3 | I | Meat | 2007 | Routine surveillance |  |
| 2928 | 1/2b | 3 | I | Meat | 2007 | Routine surveillance |  |
| 2929 | 1/2b | 3 | I | Meat | 2007 | Routine surveillance |  |
| 2940 | 1/2b, 3b | 3 | I | Dairy | 2009 | Product testing |  |
| 2947 | 1/2b, 3b | 3 | I | Dairy | 2010 | Product testing | NAUV00000000 |
| 2949 | 1/2b, 3b | 3 | I | Dairy | 2010 | Product testing |  |
| 2951 | 1/2b, 3b | 3 | I | Dairy | 2010 | Product testing |  |
| 2955 | 1/2b, 3b | 3 | I | Dairy | 2010 | Product testing | NAUY00000000 |
| 2961 | 1/2b, 3b | 3 | I | Dairy | 2011 | Product testing | NAUX00000000 |
| 2962 | 1/2b, 3b | 3 | I | Dairy | 2011 | Product testing | NAUW00000000 |
| 2971 | 1/2b, 3b | 3 | I | Dairy | 2012 | Product testing | NAUU00000000 |
| 2972 | 1/2b, 3b | 3 | I | Dairy | 2012 | Product testing | NAUT00000000 |
| 2975 | 1/2b, 3b | 3 | I | Dairy | 2012 | Product testing | NAUS00000000 |
| 2976 | 1/2b, 3b | 3 | I | Dairy | 2012 | Product testing | NAUR00000000 |
| 2982 | 1/2b, 3b | 3 | I | Dairy | 2012 | Product testing | NAUQ00000000 |
| 2984 | 1/2b, 3b | 3 | I | Dairy | 2012 | Product testing |  |
| 2992 | 1/2b, 3b | 3 | I | Dairy | 2013 | Product testing |  |
| 2996 | 1/2b, 3b | 3 | I | Dairy | 2010 | Product testing |  |
| 3005 | 1/2b, 3b | 3 | I | Dairy | 2009 | Product testing |  |
| DS_25 | 1/2b, 3b | 3 | I | Meat | 2007 | Routine surveillance |  |
| DS_68 | 1/2b, 3b | 3 | I | Meat | 2007 | Routine surveillance |  |
| DS_84 | 1/2b, 3b | 3 | I | Meat | 2007 | Routine surveillance |  |
| DS_85 | 1/2b, 3b | 3 | I | Meat | 2007 | Routine surveillance |  |
| DS_Site10 | 1/2b, 3b | 3 | I | Meat | 2007 | Routine surveillance |  |
| DS_Site13 | 1/2b, 3b | 3 | I | Meat | 2007 | Routine surveillance |  |
| DS_Site14 | 1/2b, 3b | 3 | I | Meat | 2007 | Routine surveillance |  |
| DS_Site19 | 1/2b, 3b | 3 | I | Meat | 2007 | Routine surveillance |  |
| DS_Site29 | 1/2b, 3b | 3 | I | Meat | 2007 | Routine surveillance |  |
| QH1 | 1/2b, 3b | 3 | I | Food | 2013 | Epidemiological investigation |  |
| QH10 | 1/2b, 3b | 3 | I | Food | 2013 | Product testing |  |
| QH11 | 1/2b, 3b | 3 | I | Food | 2013 | Epidemiological investigation |  |
| QH13 | 1/2b, 3b | 3 | I | Meat | 2014 | Product testing |  |
| QH15 | 1/2b, 3b | 3 | I | Food | 2014 | Epidemiological investigation |  |
| QH2 | 1/2b, 3b | 3 | I | Food | 2013 | Epidemiological investigation |  |
| QH24 | 1/2b, 3b | 3 | I | Meat | 2014 | Product testing |  |
| QH25 | 1/2b, 3b | 3 | I | Meat | 2013 | Product testing |  |
| QH26 | 1/2b, 3b | 3 | I | Meat | 2014 | Epidemiological investigation |  |
| QH27 | 1/2b, 3b | 3 | I | Meat | 2014 | Epidemiological investigation |  |
| QH29 | 1/2b, 3b | 3 | I | Environment - Food | 2014 | Epidemiological investigation |  |
| QH3 | 1/2b, 3b | 3 | I | Food | 2013 | Epidemiological investigation |  |
| QH30 | 1/2b, 3b | 3 | I | Environment - Food | 2014 | Epidemiological investigation |  |
| QH31 | 1/2b, 3b | 3 | I | Environment - Food | 2014 | Epidemiological investigation |  |
| QH34 | 1/2b, 3b | 3 | I | Meat | 2014 | Product testing |  |
| QH39 | 1/2b, 3b | 3 | I | Clinical | 2009 | Clinical case |  |
| QH4 | 1/2b, 3b | 3 | I | Food | 2013 | Epidemiological investigation |  |
| QH40 | 1/2b, 3b | 3 | I | Clinical | 2010 | Clinical case |  |
| QH41 | 1/2b, 3b | 3 | I | Clinical | 2012 | Clinical case |  |
| QH46 | 1/2b, 3b | 3 | I | Clinical | 2013 | Clinical case |  |
| QH5 | 1/2b, 3b | 3 | I | Food | 2013 | Epidemiological investigation |  |
| QH7 | 1/2b, 3b | 3 | I | Food | 2013 | Product testing |  |
| QH16 | 1/2b, 3b | 5 | I | Dairy | 2015 | Epidemiological investigation |  |
| QH43 | 1/2b, 3b | 5 | I | Clinical | 2012 | Clinical case |  |
| QH79 | 1/2b, 3b | 5 | I | Clinical | 2015 | Clinical case |  |
| 2997 | 1/2a, 3a | 7 | II | Food | 2011 | Product testing |  |
| L4935a | 1/2a | 7 | II | Animal | 1991 | Animal isolate |  |
| L4935b | 1/2a | 7 | II | Animal | 1991 | Animal isolate |  |
| L4935c | 1/2a | 7 | II | Animal | 1991 | Animal isolate |  |
| L4935d | 1/2a | 7 | II | Animal | 1991 | Animal isolate |  |
| L4938b | 1/2a | 7 | II | Environment | 1991 | Environmental sampling - feed |  |
| QH55 | 1/2a, 3a | 7 | II | Clinical | 2014 | Clinical case |  |
| L4948c | 1/2a | 8 | II | Animal | 1991 | Caprine isolate |  |
| Lm15-004 | 1/2a | 8 | II | Meat | 2015 | Routine surveillance |  |
| 2932 | 1/2c | 9 | II | Meat | 2007 | Routine surveillance | LJPE01000000 |
| DS_B2L | 1/2c | 9 | II | Meat | 2007 | Routine surveillance |  |
| DS_Site16 | 1/2c | 9 | II | Meat | 2007 | Routine surveillance |  |
| QH23 | 1/2c, 3c | 9 | II | Food | 2015 | Product testing |  |
| QH36 | 1/2c, 3c | 9 | II | Food | 2013 | Epidemiological investigation |  |
| QH42 | 1/2c, 3c | 9 | II | Clinical | 2012 | Clinical case |  |
| QH51 | 1/2c, 3c | 9 | II | Clinical | 2013 | Clinical case |  |
| QH54 | 1/2c, 3c | 9 | II | Clinical | 2013 | Clinical case |  |
| QH76 | 1/2c, 3c | 9 | II | Clinical | 2015 | Clinical case |  |
| 2998 | 1/2a, 3a | 12 | II | Meat | 2011 | Product testing |  |
| 3000 | 1/2a, 3a | 12 | II | Meat | 2011 | Product testing |  |
| 3004 | 1/2a, 3a | 12 | II | Dairy | 2009 | Routine surveillance |  |
| 3006 | 1/2a, 3a | 12 | II | Dairy | 2011 | Product testing |  |
| L4936a | 1/2a | 19 | II | Animal | 1991 | Animal isolate |  |
| L4936b | 1/2a | 19 | II | Animal | 1991 | Animal isolate |  |
| L4936c | 1/2a | 19 | II | Animal | 1991 | Animal isolate |  |
| L4936d | 1/2a | 19 | II | Animal | 1991 | Animal isolate |  |
| L4943a | 1/2a | 20 | II | Animal | 1991 | Caprine isolate |  |
| L4943b | 1/2a | 20 | II | Animal | 1991 | Caprine isolate |  |
| L4943c | 1/2a | 20 | II | Animal | 1991 | Caprine isolate |  |
| L4943d | 1/2a | 20 | II | Animal | 1991 | Caprine isolate |  |
| L4944a | 1/2a | 20 | II | Animal | 1991 | Caprine isolate |  |
| L4944b | 1/2a | 20 | II | Animal | 1991 | Caprine isolate |  |
| QH70 | 1/2a, 3a | 21 | II | Clinical | 2014 | Clinical case |  |
| Lm14-002 | 1/2a | 26 | II | Dairy | 2014 | Raw milk testing |  |
| 2987 | 1/2a, 3a | 38 | II | Dairy | 2013 | Product testing | NAUJ00000000 |
| L4941 | 1/2b | 59 | I | Animal | 1991 | Animal isolate |  |
| L4945 | 1/2b | 59 | I | Animal | 1991 | Animal isolate |  |
| L4947 | 1/2b | 59 | I | Animal | 1991 | Animal isolate |  |
| Lm14-003 | 1/2b | 59 | I | Dairy | 2014 | Raw milk testing |  |
| QH69 | 1/2b, 3b | 87 | I | Clinical | 2014 | Clinical case |  |
| L4949 | 1/2a | 91 | II | Animal | 1991 | Bovine isolate |  |
| QH72 | 1/2a, 3a | 91 | II | Clinical | 2015 | Clinical case |  |
| QH74 | 1/2a, 3a | 91 | II | Clinical | 2015 | Clinical case |  |
| 2943 | 1/2a, 3a | 101 | II | Dairy | 2009 | Product testing | NAUH00000000 |
| 2952 | 1/2a, 3a | 101 | II | Dairy | 2010 | Product testing | NAUI00000000 |
| 2974 | 1/2a, 3a | 101 | II | Dairy | 2012 | Product testing | NAUG00000000 |
| CLIP73 | 4b | 119 | I | Clinical | 1931 | Clinical case |  |
| 2884 | 1/2a, 3a | 120 | II | Seafood | 2009 | Product testing | NAUN00000000 |
| QH37 | 1/2a, 3a | 120 | II | Food | 2014 | Product testing |  |
| 2965 | 1/2a, 3a | 121 | II | Dairy | 2011 | Product testing | NAUF00000000 |
| 2985 | 1/2a, 3a | 121 | II | Dairy | 2012 | Product testing | NAUE00000000 |
| QH17 | 1/2a, 3a | 121 | II | Vegetable | 2012 | Epidemiological investigation |  |
| QH18 | 1/2a, 3a | 121 | II | Vegetable | 2012 | Epidemiological investigation |  |
| QH35 | 1/2a, 3a | 121 | II | Food | 2014 | Product testing |  |
| QH6 | 1/2a, 3a | 121 | II | Food | 2013 | Product testing |  |
| 2941 | 1/2c, 3c | 122 | II | Dairy | 2009 | Product testing | NAUP00000000 |
| 2944 | 1/2c, 3c | 122 | II | Dairy | 2009 | Product testing | NAUO00000000 |
| CLIP12 | 1/2a | 141 | II | Unknown | Pre-2008 | Unknown |  |
| SLCC3291 | 4d | 145 | I | Unknown | 1970 | Unknown |  |
| 2925 | 1/2a | 155 | II | Meat | 2007 | Routine surveillance |  |
| 2942 | 1/2a, 3a | 155 | II | Dairy | 2009 | Product testing | NAUM00000000 |
| 2946 | 1/2a, 3a | 155 | II | Dairy | 2010 | Product testing | NAUL00000000 |
| DS_Site12 | 1/2a | 155 | II | Meat | 2007 | Routine surveillance |  |
| DS_Site23 | 1/2a | 155 | II | Meat | 2007 | Routine surveillance |  |
| DS_Site25 | 1/2a | 155 | II | Meat | 2007 | Routine surveillance |  |
| DS_Site31 | 1/2a | 155 | II | Meat | 2007 | Routine surveillance |  |
| Lm15-002 | 1/2a | 155 | II | Meat | 2015 | Routine surveillance |  |
| Lm15-003 | 1/2a | 155 | II | Meat | 2015 | Routine surveillance |  |
| QH21 | 1/2a, 3a | 155 | II | Food | 2013 | Epidemiological investigation |  |
| QH22 | 1/2a, 3a | 155 | II | Seafood | 2013 | Epidemiological investigation |  |
| QH28 | 1/2a, 3a | 155 | II | Seafood | 2014 | Epidemiological investigation |  |
| QH33 | 1/2a, 3a | 155 | II | Environment - Food | 2014 | Epidemiological investigation |  |
| QH38 | 1/2a, 3a | 155 | II | Seafood | 2014 | Epidemiological investigation |  |
| QH75 | 1/2a, 3a | 155 | II | Clinical | 2015 | Clinical case |  |
| 2999 | 4a, 4c | 202 | III | Dairy | 2010 | Product testing |  |
| 2882 | 1/2a | 204 | II | Meat | 2000 | Product testing | LXQP01000000 |
| 2919 | 1/2a | 204 | II | Meat | 2007 | Routine surveillance | LXQQ01000000 |
| 2921 | 1/2a | 204 | II | Meat | 2007 | Routine surveillance |  |
| 2922 | 1/2a | 204 | II | Meat | 2007 | Routine surveillance |  |
| 2923 | 1/2a | 204 | II | Meat | 2007 | Routine surveillance |  |
| 2930 | 1/2a | 204 | II | Meat | 2007 | Routine surveillance |  |
| 2931 | 1/2a | 204 | II | Meat | 2007 | Routine surveillance |  |
| 2937 | 1/2a, 3a | 204 | II | Dairy | 2009 | Product testing | LXQR01000000 |
| 2939 | 1/2a, 3a | 204 | II | Dairy | 2009 | Product testing | LXQS01000000 |
| 2945 | 1/2a, 3a | 204 | II | Dairy | 2010 | Product testing | LXQT01000000 |
| 2964 | 1/2a, 3a | 204 | II | Dairy | 2011 | Product testing | LXQU01000000 |
| 2973 | 1/2a, 3a | 204 | II | Dairy | 2012 | Product testing | LXQV01000000 |
| 2977 | 1/2a, 3a | 204 | II | Dairy | 2012 | Product testing | LXQW01000000 |
| 2978 | 1/2a, 3a | 204 | II | Dairy | 2012 | Product testing | LXQX01000000 |
| 2981 | 1/2a, 3a | 204 | II | Dairy | 2012 | Product testing | LXQY01000000 |
| 3002 | 1/2a, 3a | 204 | II | Dairy | 2006 | Routine surveillance | LXQZ01000000 |
| DS_31 | 1/2a, 3a | 204 | II | Meat | 2007 | Routine surveillance |  |
| DS_53 | 1/2a, 3a | 204 | II | Meat | 2007 | Routine surveillance |  |
| DS_63 | 1/2a, 3a | 204 | II | Meat | 2007 | Routine surveillance |  |
| DS_88 | 1/2a, 3a | 204 | II | Meat | 2007 | Routine surveillance |  |
| DS_PRD_5 | 1/2a, 3a | 204 | II | Meat | 2007 | Routine surveillance |  |
| Lm15-001 | 1/2a | 204 | II | Meat | 2015 | Routine surveillance | LXRA01000000 |
| Lm15-011 | 1/2a, 3a | 204 | II | Meat | 2015 | Routine surveillance | LXRB01000000 |
| LM21475 | 1/2a | 204 | II | Environment | 1992 | Environmental sampling |  |
| QH12 | 1/2a, 3a | 204 | II | Food | 2014 | Epidemiological investigation |  |
| QH65 | 1/2a, 3a | 204 | II | Clinical | 2014 | Clinical case |  |
| QH8 | 1/2a, 3a | 204 | II | Environment - Food | 2013 | Epidemiological investigation |  |
| SLCC2536 | 4b | 252 | I | Clinical | 1966 | Clinical case |  |
| 2967 | 1/2a, 3a | 320 | II | Dairy | 2011 | Product testing | NAUK00000000 |
| QH14 | 1/2a, 3a | 321 | II | Meat | 2014 | Product testing |  |
| QH20 | 1/2a, 3a | 321 | II | Meat | 2014 | Product testing |  |
| QH62 | 1/2a, 3a | 321 | II | Clinical | 2014 | Clinical case |  |
| QH66 | 1/2a, 3a | 321 | II | Clinical | 2014 | Clinical case |  |
| QH67 | 1/2a, 3a | 321 | II | Clinical | 2014 | Clinical case |  |
| QH47 | 1/2b, 3b | 323 | I | Clinical | 2013 | Clinical case |  |
| QH71 | 1/2b, 3b | 324 | I | Clinical | 2014 | Clinical case |  |
| 2963 | 1/2a, 3a | 325 | II | Dairy | 2011 | Product testing |  |
| 2989 | 1/2a, 3a | 325 | II | Dairy | 2013 | Product testing |  |
| 2948 | 4b, 4d, 4e | 328 | I | Dairy | 2010 | Product testing |  |
| SLCC4319 | 4b | 354 | I | Clinical | 1975 | Clinical case |  |
| QH60 | 4b, 4d, 4e | 382 | I | Clinical | 2014 | Clinical case |  |
| 2994 | 1/2a, 3a | 480 | II | Vegetable | 2011 | Product testing |  |
| SLCC4315 | 4b | 494 | I | Clinical | 1975 | Clinical case |  |
| Lm14-001 | 1/2a, 3a | 706 | II | Environment | 2014 | Environmental sampling - water |  |
| QH64 | 1/2b, 3b | 896 | I | Clinical | 2014 | Clinical case |  |
